# Letter from London, England

**Published:** 1890-03

**Authors:** W. Mitchell

**Affiliations:** London, England


To the Editor of the Dental Register.

*Dear Sir* :—It is a hopeful sign to note that the powers that be in England, both journalistic and educational—for the time being, at least—have condescended to consider the fact that dentistry has a practical side as well as a theoretical one.

The tendency heretofore has been toward an exalted, hair-splitting theorizing that has proved about as beneficial to the profession in general as would the report of a committee upon the effect of heredity and climatic influences in the promotion of ingrowing toe nails upon the prehistoric races.

I would not be considered as not being in harmony with thorough, honest work; the more of it we have the better, but let it go hand in hand with practice, thereby evolving theory that will be founded upon a firmer basis than pure imagination; for it is well known that in dentistry, theory and practice are frequently modified in their relations, if not entirely at variance. There is also an abuse that has been creeping into the profession, *i. e.*, for men to get up at meetings and give ideas an airing. The supposed practical advantages exist only in the vaporing of their imaginations, and these utterances are allowed to go unchallenged by men of experience who know the propositions are not based upon facts.

The question now under consideration in England is how best to supplement the private prosthetic course by collegiate practical instruction, possibly somewhat after the manner now in vogue in the United States, which if carried out in an intelligent manner, can not but produce beneficial results, but if its possibilities are left to the mercies of promotors who are but slightly in touch with the requirements of the case, as is too painfully evident in many institutions in America, the effect will be the existence of an amount of "rule and thumb" that will discourage the honest student and disgust those who had hoped for the greatest resulting benefits.

In many instances in dentistry we find good men who do certain things well, when asked why they do thus and so, they reply "So and So does it that way," and that appears to be reason sufficient for them, instead of probing the subject for the pro's and con's, and giving them in an intelligent manner; this, alas, is very true of much that pertains to prosthetic dentistry, and it remains for instructors, both private and public, to remedy this defect in the system of teaching that now prevails, or what would be better, renovate the entire system.

The system in vogue in the United States, while it possesses many advantages, also has many to be considered; it is a jerky, irregular system, and one not at all calculated to develop resource, or develop the mechanical genius that is undoubtedly latent in many students; one of the greatest requirements in a dentist. This is owing in many instances to the imperfect manner in which the lectures upon the subject are delivered, and the worse surroundings of the student in the laboratory, through lack of system and ignorance in the selection and capacity of appliances.

The student is not entirely to blame for his lack of interest in this branch of our profession ; the student will, as a rule, become interested in any subject that is made interesting, and it lays almost entirely with the instructor what the results of his teaching are. All one has to do is to look over the specimen cases of graduates to see how much artistic capacity and individuality has been developed by their instructors ; the work itself, *i. e.*, the mechanical part may be well nigh perfect, but where does the individuality, the genius of the artist appear?

The majority of specimen cases handed in as part of the student's examination work are principally full upper dentures, which from the painful regularity with which the teeth are arranged, error in adaptation, whiteness, and general stereotyped appearance, I should imagine ultimately find their way into show cases of advertising quacks, there mutely, but none the less eloquently suggesting an artificiality that true art would have deftly concealed.

The advocates of the highest possibilities of dental prosthesis are but few ; these should endeavor to imbue the coming dentists with the spirit that this much-neglected branch of our profession requires; then both the professson and the public will be the gainers, and our institutions of learning will then have to live up to their name instead of only dabbling with, or shuffling around, the branch from which their profession practically springs.

Yours truly,

LONDON, ENGLAND. W. MITCHELL.

